# Role of IL-6/STAT3 Axis in Resistance to Cisplatin in Gastric Cancers

**DOI:** 10.3390/biomedicines11030694

**Published:** 2023-02-24

**Authors:** Simona Laurino, Mariarita Brancaccio, Tiziana Angrisano, Giovanni Calice, Sabino Russi, Pellegrino Mazzone, Giuseppina Di Paola, Michele Aieta, Vitina Grieco, Gabriella Bianchino, Geppino Falco, Tiziana Notarangelo

**Affiliations:** 1 IRCCS CROB, Centro di Riferimento Oncologico della Basilicata, 85028 Rionero in Vulture, Italy; 2 Department of Molecular Medicine and Medical Biotechnology, University of Naples Federico II, 80131 Naples, Italy; 3 Department of Biology, University of Naples Federico II, 80126 Naples, Italy; 4 Biogem, Istituto di Biologia e Genetica Molecolare, Via Camporeale, 83031 Ariano Irpino, Italy

**Keywords:** gastric cancer, STAT3 signalling, IL-6, cisplatin, multidrug resistance

## Abstract

Gastric cancer, the second most common cause of death worldwide, is characterized by poor prognosis and low responsiveness to chemotherapy. Indeed, multidrug resistance, based mainly on cellular and molecular factors, remains one of the most limiting factors of the current approach to gastric cancer (GC) therapy. We employed a comprehensive gene expression analysis through data mining of publicly available databases to assess the role of the signal transducer and activator of transcription 3 (STAT3) in gastric cancer drug efficiency. It has been proposed that gastric cancer cells are less sensitive to these drugs because they develop resistance to these agents through activating alternative signalling pathways responsible for overcoming pharmacological inhibition. Our study evaluated the hypothesis that activating STAT3 signalling in response to cisplatin reduces the reaction to the drug. Consistent with this hypothesis, inhibition of interleukin 6 (IL-6)/STAT3 in combination therapy with cisplatin prevented both STAT3 activation and more lethality than induction by a single agent. The data suggest that the IL-6/STAT3 axis block associated with cisplatin treatment may represent a strategy to overcome resistance.

## 1. Introduction

Gastric cancer (GC) represents the fifth most common cancer with a very poor survival outcome, ranking fourth in mortality rate [1,2].

The treatment provided in first-line therapy is based on the platinum compounds cisplatin and oxaliplatin [3], which activate apoptosis, plus fluoropyrimidine (fluorouracil, (5-FU), capecitabine, and S-1 (combination of tegafur, a prodrug of 5-FU, 5-chloro-2-4-dihydroxypyridine, and oxonic acid)) [4], which causes cell death by DNA synthesis inhibition. These are combined with docetaxel, a microtubules depolymerization inhibitor, or trastuzumab, a monoclonal antibody directed against human epidermal growth factor receptor 2 (HER2) [5]. Indeed, their clinical impact remains modest and gives an overall survival increase of only 1–3 months. A similar scenario has been reported after using second- and third-line treatments based on ramucirumab, a monoclonal antibody directed against vascular endothelial growth factor receptor 2 (VEGFR2), and the tyrosine kinase inhibitor apatinib [5,6]. Furthermore, in recent years, programmed cell death protein 1 (PD-1) or programmed death-ligand 1 (PD-L1) inhibitors have been used to manage advanced gastric cancer treatment. Unfortunately, clinical trials demonstrate that treatments with nivolumab and pembrolizumab (PD-1 inhibitors) or avelumab (PD-L1 inhibitor) show a response rate of 22–27% in terms of survival [7,8,9].

Therapy resistance reduces the effectiveness of a drug used to treat a disease. In oncology, it is the leading cause responsible for therapeutic failure at the beginning of the treatment or after an initial response to chemotherapy [10]. It is a complex, multifactorial phenomenon involving several interconnected or independent pathways that cause changes within cancer cells that become unresponsive to the treatment [11].

Cancer cells may resist a single agent or chemotherapy drugs with different structures and mechanisms of action. Therapy resistance is a significant problem when treating cancer patients because cancer cells develop mechanisms that limit the effect of therapeutic agents, selecting more aggressive clones characterized by poor prognosis. The resistance can be primary or intrinsic and secondary or acquired if it occurs, respectively, before or after exposure to a chemotherapy drug. Indeed, the multidrug-resistance (MDR) phenotype is associated with resistance to a broad spectrum of drugs and correlates with an aggressive phenotype and negative follow-up [12,13].

The potential mechanisms of MDR currently include drug efflux through ATP-binding cassette (ABC) transporters, acquired mutations in ligands or receptors, evading apoptosis, activation of the DNA damage response, induction of autophagy, regulation of cancer stem cells, miRNA regulation, induction of hypoxia, and epigenetic regulation [13].

In this view, cancer stem cells (CSCs), a subset of tumour cells capable of self-renewal known to be intrinsically resistant to anticancer treatments, represent the primary source of therapy resistance [14]. Multiple clones of CSCs pre-exist, and some can adapt and expand quickly to changes in the tumour microenvironment (TME) and/or in response to chemotherapy. A combination of intrinsic and extrinsic factors contributes to CSC-mediated therapy resistance.

Chemoresistance in gastric cancer represents a significant limitation responsible for unsatisfactory clinical outcomes and the high mortality rate of patients with gastrointestinal cancers [14,15,16]. Cancer cells may develop multiple mechanisms of drug resistance, mainly through apoptosis inhibition, abnormal drug activation, and an altered mechanism of cellular extrusion of the drug [13].

In such a context, the unresponsiveness to conventional therapy and the development of the metastatic phase correlates with biologically and clinically aggressive features. Therefore, we investigated the mechanisms underlying cancer drug resistance to identify likely targets and improve the efficacy of chemotherapy. From a biological perspective, several oncogenic alterations have been described in human gastric cancer, which are responsible for cell cycle progression, the activation of angiogenetic mechanisms, and the evasion of apoptotic pathways that confer a survival advantage. Indeed, GC cell lines derived from metastatic sites are poorly sensitive to treatment with some antineoplastic agents (e.g., mitotic inhibitors) due to the resistance acquired by activating alternative signalling pathways [2,17,18,19]. In this scenario, the role of the signal transducer and activator of transcription 3 (STAT3) pathway is poorly understood in gastric cancer. Still, it has been described as a mediator of tumorigenesis in several human malignancies, being overactive in many tumour types, including all major carcinomas and some hematologic tumours [20,21,22]. Indeed, literature data suggest a crucial role of the aberrant, hyperactivated STAT3 pathway in driving drug resistance to vemurafenib in melanoma cells [23,24,25]. Moreover, in head and neck squamous carcinoma cells, NVP-BKM120, a pan-PI3K inhibitor, leads to upregulation of IL-6 and subsequent activation of either ERK or STAT3 signalling, as well as expression of the *MYC* oncogene, and this axis mediates adaptive resistance to PI3K inhibition [26,27]. Furthermore, recently, a role of activated interleukin 6 (IL-6)/STAT3 signalling has been reported in driving the resistance of BRAF-mutated thyroid cancer cells to vemurafenib, and the blockade of the IL-6 or STAT3 axis has been proposed to increase vemurafenib activity [28].

In recent years, inflammatory mechanisms have been proposed as an essential player in tumour pathogenesis [29,30]. Among other factors, several cytokines regulate the inflammatory tumour microenvironment and change the response to chemotherapeutic agents. For example, literature data suggest that treatment with PD-1 or PDL-1 inhibitors induces the release of cytokines, generating an inflammatory tumour microenvironment and reducing the efficacy of chemotherapeutic agents [7,31,32,33,34]. Cytokines involved in cancer-related inflammation represent a target for innovative therapeutic strategies [34]. Indeed, several inflammatory cytokines regulate cancer cell growth, contribute to tumour progression, and escape to apoptosis [27,35]. Among these, IL-6 is associated with an unfavourable prognosis in patients with various solid and haematological diseases [26,29,36]. Indeed, other authors reported that the STAT3 pathway is activated in response to stimulation of the heterodimeric GP130/IL-6 cytokine-specific receptor complex in melanoma cells [23,25,37,38]. The activated STAT3 pathway, in response to the binding of IL-6 secreted to its glycoprotein 130 (GP130) receptor, promotes tumour progression through the induction of various target genes involved in tumour cell survival, proliferation, angiogenesis, metastasis, and cell adhesion. Consequently, in oncology, IL-6/STAT3 is the target of several therapies. IL-6 and STAT3 inhibition may represent a likely target that could revert acquired resistance [39]. In this context, increased IL-6 secretion, together with the upregulation of GP130, the IL-6 receptor, is likely responsible for increased STAT3 expression and poor sensitivity to treatment with chemotherapeutic agents. Similarly, it is interesting to hypothesize that IL-6 expression/secretion is probably enhanced by the early upregulation of CEBPβ, a transcription factor responsible for the modulation of several genes involved in immune and inflammatory responses and, among others, IL-6 [20].

Therefore, our study aimed to identify, as likely therapeutic targets, the IL-6 secretion and the consequent STAT3 activation driving drug resistance. In order to disclose these therapeutic targets, we performed: (a) a deep bioinformatic analysis of public datasets to evaluate STAT3 expression levels in healthy and GC patients; (b) an evaluation of *STAT3* gene and protein expression levels using two GC cell lines (metastatic KATO III and primary SNU-1) in the absence and in the presence of cisplatin; (c) analysis of the gene expression of *IL-6* and *CEBPβ* in KATO III subjected to treatment with cisplatin; (d) ELISA assays to measure IL-6 secretion in KATO III cells following exposure to cisplatin; (e) apoptotic assays to monitor cell death following treatment with different therapeutic agents, namely, cisplatin, HO3867 (STAT3 inhibitor), and tocilizumab (anti-IL-6 receptor).

Overall, we highlight a possible role of IL-6/STAT3 as targets for the treatment of GC, likely in combination with conventional chemotherapeutics to enhance their effectiveness.

## 2. Materials and Methods

### 2.1. Bioinformatics Analysis

Dataset GSE100935 was downloaded from the GEO public repository and investigated for genes of interest. To evaluate the set of normal patients, as a control, datasets GSE13861 and GSE66229 were downloaded from GEO and composed together, and the batch effect was removed by the limma package common function [40].

Custom boxplots were implemented by the ggpubr package (https://cran.r-project.org/web/packages/ggpubr/, accessed on 7 March 2022). The Wilcoxon rank sum test evaluated the distribution difference [41].

All analyses were performed by the R/Bioconductor environment (https://www.r-project.org; https://www.bioconductor.org, accessed on 7 March 2022) [42].

### 2.2. Chemicals

Unless otherwise specified, reagents were purchased from Sigma-Aldrich (Milan, Italy). STAT3 inhibitor, HO-3867 (Cat. No S7501), was purchased from Selleck Chemicals (Houston, TX, USA). Drugs were dissolved in dimethylsulfoxide (DMSO), and the same DMSO volume was added to the untreated control. IL-6 was purchased from Miltenyi Biotec (Bologna, Italy).

Cisplatin was obtained from Accord Healthcare Limited (Harrow, UK).

### 2.3. Cell Cultures

KATO III (RRID: CVCL_0371) and SNU-1 (RRID: CVCL_0099) gastric cancer cell lines were purchased from ATCC (Manassas, VA, USA) and were cultured in Iscove’s Modified Dulbecco’s Medium (IMDM, GIBCO, Grand Island, NY, USA) containing 20% (*v*/*v*) foetal bovine serum (FBS, GIBCO, Grand Island, NY, USA) and 100 U/mL penicillin and streptomycin (GIBCO (Grand Island, NY, USA) and Roswell Park Memorial Institute (RPMI, GIBCO, Grand Island, NY, USA)) containing 10% (*v*/*v*) foetal bovine serum (FBS) and 100 U/mL penicillin and streptomycin, respectively, according to manufacturer instructions. GC cells were incubated at 37 °C in 5% CO_2_; the medium was changed daily, and cells were split routinely every 2–3 days. The GC cancer cell lines originated from primary (SNU-1) and metastatic sites (KATO III).

### 2.4. Apoptosis Assay

The KATO III and SNU-1 cell lines were exposed to cisplatin (10 µM) for 48 h to assess their sensitivity. Similarly, to evaluate the effect of STAT3 blockade by HO3867 on the cisplatin resistance of cell lines, cisplatin (10 µM) was added in concomitant exposure to HO3867 (10 µM).

After 48 h of culture, KATO III and SNU-1 cells were recovered by trypsinization and subjected to flow cytometry. In brief, cells were washed twice with cold PBS and then, after centrifugation, resuspended in 100 µL of 1X binding buffer at a concentration of 1 × 10^6^ cells/mL. Subsequently, 5 µL of FITC Annexin V and 5 µL propidium iodide (PI) (BD Biosciences, San Jose, CA, USA) were added, and cells were incubated for 15 min at room temperature (RT) in the dark. For each tube, an adequate volume of 1X binding buffer was added. All samples were acquired, within 1 h, by using a NAVIOS flow cytometer and analysed by Kaluza software (Beckman Coulter Diagnostics, Brea, CA, USA). A total of 10,000 events were acquired for each sample. Data from treated samples were normalized as the fold change of untreated controls and reported as the mean ± SD (standard deviations) of at least three independent experiments.

### 2.5. Western Blot Analysis

Total cell lysates were obtained by the homogenization of cell pellets and surgical specimens in a cold lysis buffer (20 mmol/L Tris, pH 7.5 containing 300 mmol/L sucrose, 60 mmol/L KCl, 15 mmol/L NaCl, 5% (*v*/*v*) glycerol, 2 mmol/L EDTA, 1% (*v*/*v*) Triton X-100, 1 mmol/L PMSF, 2 mg/mL aprotinin, 2 mg/mL leupeptin, and 0.2% (*w*/*v*) deoxycholate) for 2 min at 4 °C and further sonication for 30 s in ice.

According to the manufacturer’s instructions, protein concentration was determined using the Bio-Rad protein assay kit (Bio-Rad, Hercules, CA, USA). Thirty-five micrograms of protein were separated on 4–20% Criterion TGX Precast Gels (Bio-Rad), according to Laemmli. Following electrophoresis, proteins were transferred onto a polyvinylidene difluoride (PVDF, Bio-Rad) membrane (Bio-Rad Trans-Blot turbo system).

These were incubated overnight at 4 °C with the following primary antibodies diluted in Phosphate-buffered saline (PBS) 1X, according to the different experiments: rabbit polyclonal anti-human STAT3 (1:1000, Santa Cruz Biotechnology, CA, USA, cat no. sc-482), rabbit monoclonal anti-human IL-6 (1:1000, Cell Signaling Technology, Denver, MA, USA, cat no. 12153), rabbit polyclonal anti-human GP130 (1:1000, Cell Signaling Technology, cat no. 3732), rabbit polyclonal anti-human CEBPβ (1:1000, Cell Signaling Technology, cat no. 3082), and as an internal control, mouse monoclonal anti-human GAPDH (1:1000 Santa Cruz Biotechnology, cat no. sc-47724). After thorough PBS washing, blots were incubated with HRP-conjugated mouse anti-rabbit IgG (1:5000, Cell Signaling Technology) diluted in PBS 1X. Protein bands were revealed by Clarity Western ECL Substrate and ChemiDoc System (Bio-Rad). Densitometry analyses were performed with ImageJ.

### 2.6. RNA Extraction and cDNA Synthesis

Total RNA was extracted using RNeasy Kits (Qiagen, Hilden, DE). The amount of total extracted RNA was estimated by measuring the absorbance at 260 nm and the purity by 260/280 and 260/230 nm ratios by Nanodrop (Thermo Fisher Scientific, Waltham, MA, USA). For each sample, 1000 ng of total RNA was retrotranscribed using the First Strand cDNA Synthesis Kit (Roche, Basilea, CH).

### 2.7. Real Time

For Real-Time PCR (qPCR) analysis, 0.5 ng of cDNA sample was amplified using the Light-Cycler 480 SYBRGreen I Master (Roche) in a Light Cycler 480 (Roche). Reaction conditions were as follows: preincubation at 95 °C for 5 min, followed by 45 cycles of 10 s at 95 °C, 10 s at 60 °C, and 10 s at 72 °C. The specific primers used for amplification were designed to be intron-spanning and are reported in Table 1. *β-Actin* was chosen as an internal control.

Calculations of relative expression levels were performed using the 2^−ΔΔCt^ method [43]. All analyses were conducted in triplicate to guarantee the accuracy of the results.

### 2.8. IL-6 Detection by ELISA Assay

IL-6 concentration was determined by the in vitro enzyme-linked immunosorbent assay IL-6 ELISA kit (Thermo Fisher Scientific, cat. no EH2IL-6), as reported in the manufacturer’s instructions.

### 2.9. Statistical Analysis

All statistical analyses were performed using GraphPad Prism 8.0.1 (GraphPad Software Inc., La Jolla, CA, USA). Data were expressed as the means ± SD. As appropriate, comparisons among groups were made by Student’s t-test or analysis of variance ANOVA, followed by Dunnett’s multiple comparison tests. Values of *p* < 0.05 were considered significant.

## 3. Results

### 3.1. STAT3 Upregulation in Patients Affected by Gastric Cancer Treated with Cisplatin/Oxaliplatin

To establish the sensitivity of GC tumours to platinum-based chemotherapy drugs (e.g., cisplatin or oxaliplatin), we evaluated the STAT3 expression level using a gene expression profiling dataset generated by Yong et al. [40]. In this study, the authors performed a gene expression profiling of 81 patients, divided into 3 cohorts of 48 human G-intestinal (G1), 21 G-diffuse (G2), and a third group (G3) of 12 unspecified tumours. The patients related to subgroups G1, G2, and G3 were treated with cisplatin/oxaliplatin, plus S-1, cisplatin plus S-1, and oxaliplatin, respectively. The features of patients under treatment are reported in Supplementary Table S1.

We noticed that STAT3 is characterized by a medium/high expression level in the whole dataset, so subsequently, we opted to evaluate its expression level in a set of normal patients used as a control. In particular, we obtained the group of normal patients from two datasets deposited on GEO, identified by the GSE13861 and GSE66229 accession numbers. We realized a significant difference between the previously evaluated STAT3 expression level and a normal one, reported together.

**Figure 1:**
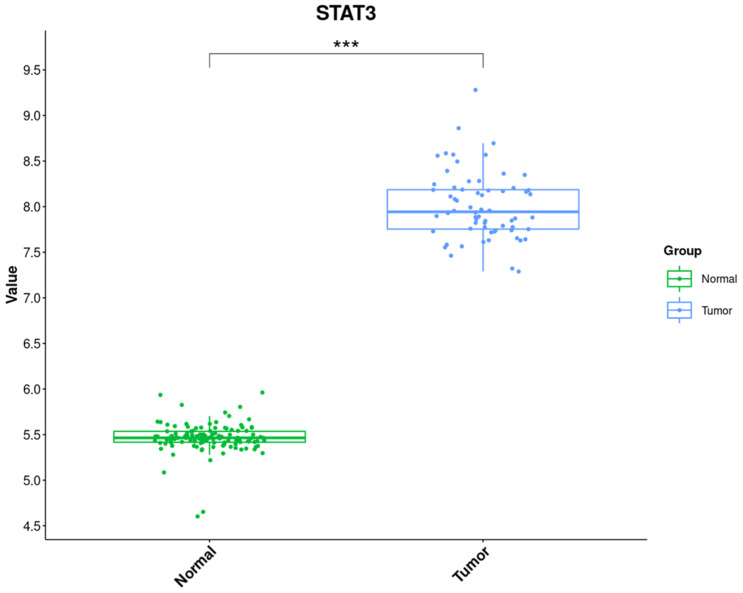
Gene Expression Level of the Tumour Samples group and Normal Samples deposited on the GEO dataset. The data show a statistically significant variation between the two groups, Normal and Tumour. The Wilcoxon rank sum test determined the significance, *** *p*-value < 0.001.

The data support the hypothesis that the STAT3 upregulation expression level difference between the two groups, tumour vs normal, is a molecular feature involved in the GC samples’ response to platinum-based chemotherapy drugs (Figure 1).

### 3.2. STAT3 Expression Is Upregulated in Human Gastric Cancer Cell Lines Derived from Metastatic Sites Exposed to Cisplatin

**Figure 2:**
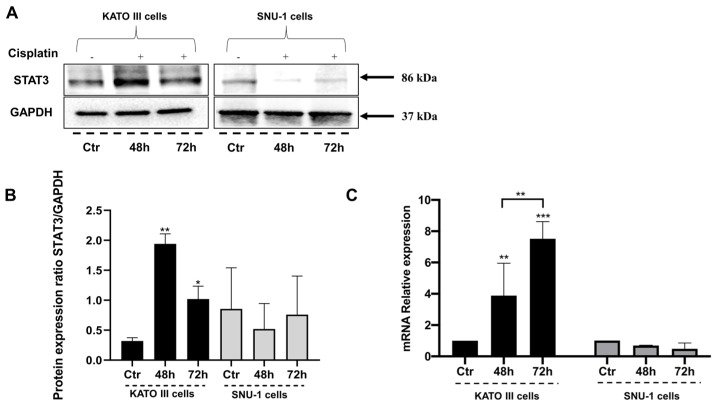
STAT3 is upregulated in human gastric cancer cells exposed to cisplatin. (**A**) A representative experiment of western blot analysis from KATO III and SNU-1 cells exposed to cisplatin for 48 and 72 h; Ctr (control cells). (**B**) STAT3 densitometric analysis; Ctr (control cells). Data were normalized for GAPDH. The data are expressed as the means ± SD. The significance was determined by one-way ANOVA followed by Dunnett’s multiple comparison tests. * (*p* < 0.05), ** (*p* < 0.01). (**C**) qPCR of *STAT3* gene expression in KATO III and SNU-1 cells exposed to cisplatin for 48 and 72 h; Ctr (control cells). The significance was determined by two-way ANOVA followed by Dunnett’s multiple comparison tests. Ctr (control) referred to untreated cells; ** (*p* < 0.01), *** (*p* < 0.001).

To confirm the involvement of STAT3 upregulation in response to cisplatin in human gastric cancer cell lines derived from the metastatic site, the protein level of STAT3 was evaluated in KATO III and SNU-1 cells exposed to 10 μM of cisplatin for 48 and 72 h by western blot analysis (Figure 2A,B). The densitometric analysis revealed a significant increase of STAT3 in KATO III cells after exposure to cisplatin for 48 and 72 h (Figure 2A,B). At the same time, we did not see any significant variation in SNU-1 cells (Figure 2A,B). Moreover, to verify that the treatment of cisplatin could influence both the STAT3 protein and gene expression, we performed a gene expression analysis by qPCR in the same conditions (Figure 2C). The qPCR analysis confirmed a significant increase of STAT3 gene expression induced by cisplatin for 48 and 72 h of treatment, specifically in KATO III cells, compared to control cells (Figure 2C).

### 3.3. Combined Treatment with Cisplatin and STAT3 Inhibitor Enhances GC Cell Response to Cisplatin

**Figure 3:**
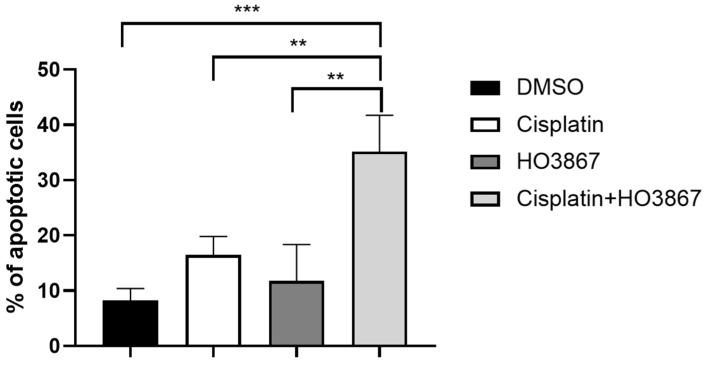
Combined treatment with cisplatin and STAT3 inhibitor enhances the apoptotic rate compared to cisplatin. Apoptotic cell death in KATO III gastric cancer cell lines exposed to 10 μM of cisplatin or 10 μM of HO3867 (STAT3 inhibitor) or the combination of both agents for 48 h. The data are expressed as the means ± SD. The significance was determined by one-way ANOVA followed by Dunnett’s multiple comparison tests. ** (*p* < 0.01), *** (*p* < 0.001).

To investigate the role of hyperactivated STAT3 in resistance to cisplatin in GC cells, we hypothesized that the blockade of STAT3 by a specific inhibitor could potentiate the activity of cisplatin. The cytotoxic activity of combined therapy with cisplatin and STAT3 inhibitor, HO3867, was evaluated compared to cisplatin and HO3867 single agents in KATO III cells. While cisplatin and HO3867 single agents showed minimal cytotoxic activity, the exposure of KATO III cells to combination therapy with both drugs resulted in a fourfold induction of apoptotic cell death (Figure 3).

### 3.4. The Exposure to Cisplatin Induces a Modulation of the JAK/STAT Pathway in GC Cells

**Figure 4:**
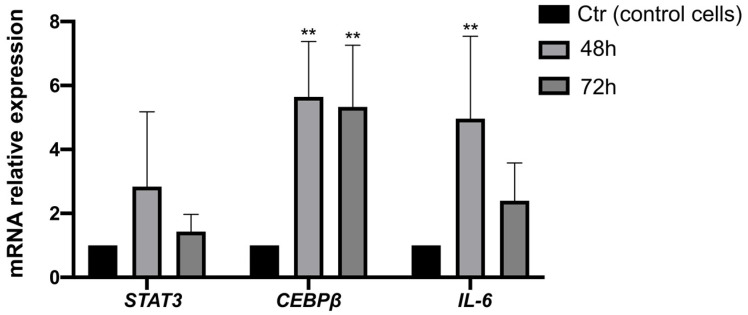
Exposure to cisplatin results in the modulation of STAT3-related genes in gastric cancer cells. Gene expression analysis of STAT3, IL-6, and CEBPβ genes in KATO III cells exposed to cisplatin for 48 and 72 h by qPCR analysis. The data are expressed as the means ± SD. The significance was determined by one-way ANOVA followed by Dunnett’s multiple comparison tests: ** (*p* < 0.01).

To investigate the mechanism of STAT3 activation, we further evaluated, in KATO III cell lines exposed to 10 µM of cisplatin, the expression of STAT3 pathway-related genes such as *IL-6*, *CEBPβ,* and transcription factors responsible for the gene regulation involved in the inflammatory response. The data suggest a modulation of the whole pathway, consistent with our hypothesis. Furthermore, in agreement with the literature data, there is an upregulation of *IL-6* expression, likely responsible for STAT3 activation in response to cisplatin (Figure 4).

### 3.5. IL-6 Secretion Mediates STAT3 Upregulation in Response to Cisplatin

To investigate the mechanism of STAT3 activation, we used the data obtained by a preformatted gene pathway array that showed, consistent with bibliographic evidence, a modulation of IL-6 and its receptor. Indeed, literature data suggested that the STAT3 pathway is activated upon tumour cell stimulation with IL-6.

**Figure 5:**
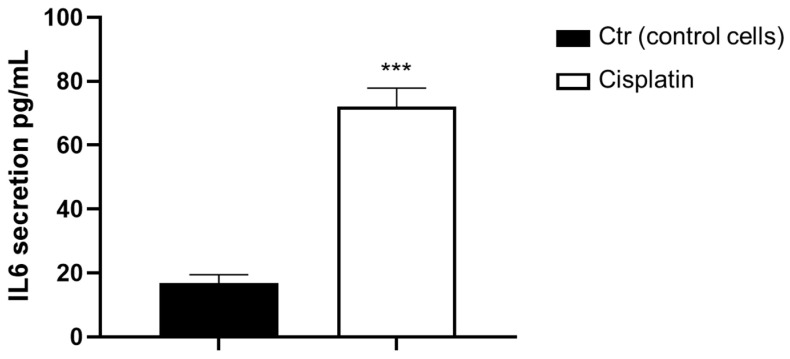
IL-6 secretion mediates STAT3 upregulation in GC cells. IL-6 levels in KATO III cells exposed to cisplatin for 48 h. The data are expressed as the means ± SD. The student’s *t*-test determined the significance: *** (*p* < 0.001) represents significance compared to control.

Therefore, IL-6 secretion was evaluated by ELISA assay in KATO III cells exposed for 48 h to 10 µM of cisplatin. The data confirmed that the treatment with cisplatin induces significant IL-6 secretion after 48 h of treatment, with a fivefold increase in the amount of IL-6 secreted (Figure 5).

**Figure 6:**
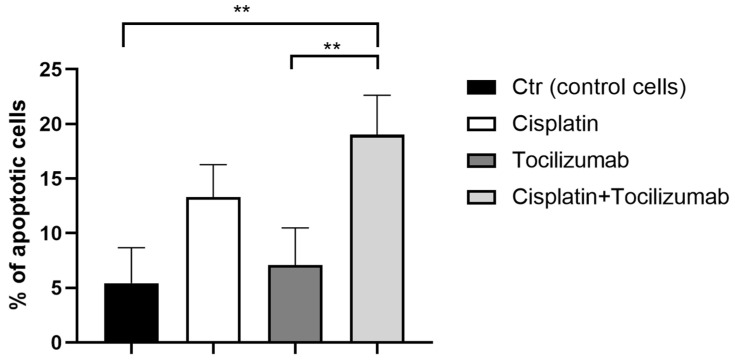
Tocilizumab combined with cisplatin induces a significant apoptotic effect. Apoptotic cell death in KATO III GC cell lines exposed to 10 μM of cisplatin or 50 mg/mL of tocilizumab or the combination of both agents for 48 h by apoptotic assay. The data are expressed as the means ± SD. The significance was determined by one-way ANOVA followed by Dunnett’s multiple comparison tests: ** (*p* < 0.01).

To confirm the hypothesis that IL-6 targeting may provide a strategy to potentiate GC cells’ response to cisplatin, we tested combination therapy with cisplatin and the anti-IL-6 receptor antibody, tocilizumab. Interestingly, while tocilizumab did not show any cytotoxic activity as a single agent, the combined exposure to tocilizumab and cisplatin resulted in a more apoptotic effect than cisplatin alone (Figure 6).

## 4. Discussion

Recent years have highlighted the role of the inflammatory tumour microenvironment in mediating the efficacy of chemotherapeutic agents [31,33].

The molecular functions of STAT3 have been extensively discussed in malignant tumours, mainly including its influence on the cell cycle, inflammatory process, and angiogenesis [21].

Noticeably, the activation of STAT3 signalling is frequent in several tumours. In this scenario, the role of the IL-6/STAT3 pathway may represent a target for innovative gastric cancer therapeutic strategies [28,33,34,35] often associated with clinically aggressive features and resistance to conventional therapy [44,45,46].

Moreover, some authors indicated an association between the phosphorylation of STAT3 (p-STAT3) and GC prognosis, as well as the clinical pathological characteristics of patients. In particular, it was demonstrated that in patients with GC, increased p-STAT3 expression is predictive of poor prognosis because it is functionally associated with worst cancer differentiation and positive lymph node metastasis [47,48,49].

Although literature data consistently indicate STAT3 as a biomarker predicting poor prognosis of GC, research has poorly investigated its role in cancer treatment efficacy. Cytokines and growth factors secreted in cancer cells can lead to persistent activation of STAT3 and, consequently, to tumorigenesis. Among cytokines, based on its association with drug resistance, IL-6 could be a relevant therapeutic target [50,51].

Based on this rationale, we evaluated the hypothesis that IL-6/STAT3 inhibition in combination with cisplatin could prevent STAT3 activation, which is associated with a better prognosis.

First, we identified STAT3 upregulation through a bioinformatic analysis of available databases in human gastric patients treated with platinum compound compared to normal. According to this, we validated the in silico data in GC cells, human KATO III, after exposure to cisplatin, observing *STAT3* protein and gene expression.

Second, using HO3867, a STAT3 inhibitor, we demonstrated the direct role of STAT3 in cisplatin resistance. The treatment combination of cisplatin and HO3867 increased the apoptosis process in the KATO III cell line.

Then, we observed that in GC cells, cisplatin-treated STAT3 expression was related to *IL6* and *CEBPβ* gene induction. *IL6* and *CEBPβ* genes belong to the STAT3 pathway.

Overall, our data suggested that cisplatin treatment induced the activation of the IL-6/STAT3 axis, with increased secretion of IL-6, which is responsible for STAT3 upregulation and therapy resistance.

Consistently, we characterized the expression levels of 84 key genes involved in the activation of the STAT3 downstream pathway in KATO III cells treated with cisplatin, HO3867, and their combination. The profiling allowed the identification of several genes significantly modulated and involved in the JAK/STAT3 pathway, such as *IL-6*. Therefore, we confirmed that cisplatin treatment determined IL-6 secretion, which is likely correlated with STAT3 upregulation [26].

In this view, lastly, we evaluated the effect of the blockade of IL-6 by a specific inhibitor (tocilizumab) in combination therapy with cisplatin on the apoptotic rate compared to the control. Our data confirmed the hypothesis related to IL-6 as a therapeutic target in cancer marked by STAT3 expression and cisplatin resistance.

## 5. Conclusions

The data in our possession highlighted the IL-6/STAT3 signalling as a target to prevent/disrupt the molecular mechanisms responsible for poor response to cell signalling inhibitors in solid tumours and as a candidate for tocilizumab as a new therapeutic agent.

In conclusion, from a clinical perspective, our study, on the one hand, deserves further confirmation by in vivo studies and by clinical trials. On the other hand, our data point to STAT3 blockade as a potential strategy to restore/delay cisplatin resistance in GC cell models.

## Figures and Tables

**Table 1:** List of genes and sequences of primers used for qPCR analysis.

Gene	Primer for 5′-3′	Primer Rev 5′-3′
*IL-6*	CTAGATGCAATAACCACCCC	CAACAACAATCTGAGGTGC
*STAT3*	GTGAGGCAGAACAGCTAGAG	GTCGTCTCCCCCTTAATTC
*CEBP* *β*	CTCTGCTTCTCCCTCTGC	CCCGTAGGAACATCTTTAAGC
*β-Actin*	GACAGGATGCAGAAGGAGAT	TTGCTGATCCACATCTGCTG

## Data Availability

Data is contained within the article and supplementary materials.

## Supplementary Materials

The following supporting information can be downloaded at: https://www.mdpi.com/article/10.3390/biomedicines11030694/s1, Supplementary Table S1: Baseline characteristics.
