# Supplementary material for: Role of IL-6/STAT3 Axis in Resistance to Cisplatin in Gastric Cancers
Source: Biomedicines. 2023 Feb 24;11(3):694. doi: 10.3390/biomedicines11030694 (PMC10044743; doi:10.3390/biomedicines11030694)
Supplement: Supplementary file 1 [file biomedicines-11-00694-s001.zip › biomedicines-2200667-supplementary.pdf]

Supplementary Table S1. Baseline characteristics.

	GSE100935	GSE13861	GSE66229
**N. Samples**	66	19	100
**Gender (F/M)**	30/36		
**Age (Median)**	58		
**Race**	23 Chinese<br>37 Korean<br>4 Malay<br>1 Myanmese<br>1 Sikh		
**Histological Type**	29 Adenocarcinoma<br>4 Diffuse<br>4 Intestinal<br>8 Signet ring cell carcinoma<br>21 NA or Not reported		
**Histological Grade**	8 Moderately differentiated<br>1 Well differentiated<br>29 Poorly differentiated<br>28 NA		
**Overall Response**	22 Partial Response<br>30 Stable Disease<br>8 Progressive Disease<br>6 NA		
